# Patient-Reported and Physiologic Markers of Pain and Anxiety during High-Resolution Manometry: An Exploratory Study

**DOI:** 10.1159/fpl/acvag016

**Published:** 2026-09-10

**Authors:** Sarah Stewart, James A. Curtis, Valentina Mocchetti, Ilan Palte, Felice Schnoll-Sussman, Amir Soumekh, Rasa Zarnegar, Philip Katz, Anaïs Rameau

**Affiliations:** a Division of Gastroenterology, Department of Medicine, Weill Cornell Medicine, New York, NY, USA; b Sean Parker Institute for the Voice, Department of Otolaryngology – Head and Neck surgery, Weill Cornell Medicine, New York, NY, USA; c Department of Surgery, Weill Cornell Medicine, New York, NY, USA

**Keywords:** Dysphagia, Laryngology, Manometry, Pain

## Abstract

**Introduction:**

Although HRM is widely considered a well-tolerated and minimally invasive procedure, pain and anxiety are reported by patients. We aimed to investigate whether patient-reported and physiologic indicators of procedure-related pain and anxiety are associated with high-resolution manometry (HRM) findings at rest and during swallowing.

**Methods:**

HRM data and patient-reported measures and physiologic indicators of pain and anxiety were collected from 36 outpatient adults. Multivariable linear regression models were used to assess the associations between pain/anxiety and upper esophageal sphincter (UES)/lower esophageal sphincter pressures and esophageal motility outcomes. Predictor variables included (1) a validated visual analogue scale (VAS) of self-reported pain; (2) the State Trait Anxiety Inventory (STAI) sum score; (3) the Short-Form McGill Pain Questionnaire (SFMPQ); (4) heart rate before the procedure (HR); and (5) average slope of galvanic skin response (GSRslope). Relationships were assessed both at rest for basal measures and during swallowing for peristaltic measures.

**Results:**

Few statistically significant relationships were observed between patient-reported measures or physiologic indicators of anxiety/pain with esophageal or sphincter measures. However, UES mean residual pressure during swallowing was negatively associated with VAS pain ratings (*p* = 0.04) and the GSRslope (*p* = 0.01). Additionally, as SF-MPQ increased, the percentage of intact swallows decreased (adjusted R^2^ = 0.146; *p* = 0.012).

**Conclusion:**

UES resting pressure during HRM procedures was not significantly associated with patient-reported anxiety or pain. However, we saw a small statistically significant inverse association between UES mean residual pressure and patient-reported pain and physiologic arousal (GSRslope). Future research may benefit from continuous physiologic monitoring throughout HRM procedures and from inclusion of a larger sample with more diverse pathology.

## Introduction

High-resolution manometry (HRM) is the clinical reference standard for quantifying pharyngeal and esophageal pressures during swallowing in people with dysphagia [1–4]. This procedure involves transnasal passage of a flexible, solid-state, pressure-sensing catheter through the pharynx, upper and lower esophageal sphincters, and into the stomach.

Although HRM is widely considered a well-tolerated and minimally invasive procedure, pain and anxiety are reported by patients [1, 5–7]. This is important because emotional state and acute stress have been found to influence esophageal and lower esophageal sphincter pressures, potentially influencing HRM results and negatively affecting diagnostic accuracy, clinical impressions, and treatment recommendations related to swallow function [8–13]. Notably, previous research has yielded inconsistent directionality and degree of findings regarding which esophageal and sphincter pressure measures are most sensitive to stress and procedural factors during HRM, underscoring the importance of considering patient-reported measures and physiologic indicators [8–13]. This inconsistency underscores the complexity of interpreting HRM measures in the context of patient stress and discomfort and the importance of characterizing that stress with validated tools: patient-rated scales such as the visual analog scale and the State-Trait Anxiety Inventory, and physiologic indicators such as heart rate and galvanic skin response, are widely used to assess pain and anxiety during medical procedures [14–17].

The aim of this study was to assess the relationship between patient baseline anxiety immediately prior to HRM and esophageal, upper esophageal sphincter (UES), and lower esophageal sphincter (LES) pressures at rest and during swallowing. Additionally, we explored how patient-reported discomfort during HRM may influence UES and LES pressures and overall esophageal motility. We hypothesized that higher levels of patient-perceived and physiologic indicators of pain and anxiety would positively correlate with increased esophageal and sphincter pressures, reflecting a physiological stress response that may influence diagnostic accuracy and interpretation of HRM findings.

## Methods

### Study Design and Participants

This study was a secondary analysis of prospectively collected data from 40 patients with dysphagia who participated in a randomized clinical trial examining the effects of a virtual reality (VR) headset-based meditation on patient-reported discomfort and anxiety during the HRM procedure described by Palte et al., 2024. This study was approved by the Institutional Review Board at Weill Cornell Medicine (IRB 20-01021300-16), and all participants provided informed consent prior to participation. English-speaking patients were recruited from the otolaryngology, gastroenterology, and general surgery clinics at Weill Cornell Medicine/New York-Presbyterian Hospital and underwent HRM for swallow function assessment. Participants were consecutive adult patients referred for HRM for evaluation of dysphagia from the otolaryngology, gastroenterology, and general surgery clinics as part of routine clinical care. The diagnosis of dysphagia was established by the referring clinician within each specialty; no standardized instrumental swallowing assessment or patient-reported dysphagia measure was uniformly performed across the cohort prior to HRM. Patients were excluded if they used psychotropic or analgesic medication within 12 h of HRM. More details about the protocol can be found in our previously published study [18].

Analyses were completed on 36 of 40 patients with complete data. The analyses focused on the relationship between HRM outcomes and independent variables including baseline state anxiety, physiologic stress indicators during manometer insertion, and self-reported pain during the procedure.

### Patient Emotional Indicators of Pain and Anxiety

The primary independent variables included patient-reported pain and anxiety and physiologic metrics. Before initiating the HRM protocol, anxiety was assessed using the State-Trait Anxiety Inventory (STAI-6), a validated score ranging from 6 (least anxious) to 24 (most anxious) [18, 19].

Following HRM completion, participants completed the Short-Form McGill Pain Questionnaire (SF-MPQ), which within it includes a visual analog scale (VAS), to assess pain quality and intensity. Patients were asked to focus their ratings on pain experienced during manometry insertion, identified as the most uncomfortable portion of the procedure [1]. The SF-MPQ includes 15 questions, each rated on a 4-point Likert-scale (i.e., throbbing, shooting, aching, etc.) from “none” to “severe” with a total composite score ranging from 0 (no pain) to 45 (severe pain). The VAS ranges from 0 (no pain) to 100 (worst possible pain) [20].

During catheter insertion, physiologic responses were measured including heart rate and galvanic skin response (GSR) (GSR Amp FE116, ADInstruments LTD, Oxford, UK). Heart rate was measured prior to the start of the procedure with a pulse transducer (TN1012/ST; PowerLab, ADInstruments LTD, Oxford, UK) and is reported as HR before (HR). GSR was measured throughout the entire procedure as a marker of autonomic nervous system activation. The maximum slope of GSR conductance (GSRslope) was calculated to reflect rate of physiologic stress, as described in Palte et al. [18], which may be indicative of pain [21]. Figure 1 summarizes the timing of data collection throughout the study.

**Fig. 1.**
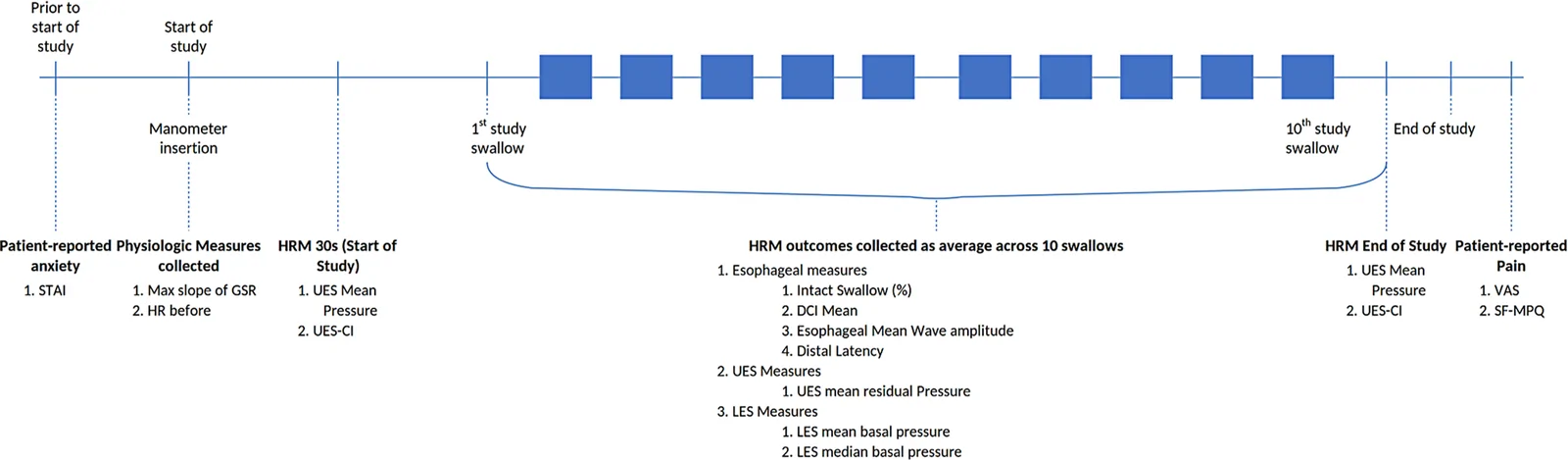
Study design.

### HRM Esophageal Measures

All patients underwent HRM after the application of a standard analgesia protocol (flushing of nostrils with 6 mL of 2% viscous lidocaine). Standard solid-state catheters with 4.2-mm outer and 36 circumferential sensors at 1-cm intervals were used (Medtronic). Following catheter insertion, subjects had a 1–3-min rest period before beginning the swallowing assessment in alignment with Chicago Classification 4.0 © standard protocol, which includes a > 60 s adaptation period for both supine and upright swallows [22]. According to protocol, ten 5-mL wet swallows in the supine position were given to the patient, each separated by at least 30 s. Esophageal and LES metrics are reported as averages across these ten swallows. UES landmark measures were captured in the supine position at the beginning of the study and in the upright position at the end of the study, immediately prior to catheter removal. Medtronic ManoView ESO 3.3 software was utilized for analysis.

Initially, the patient’s average tidal breath duration was determined by measuring the duration (seconds) of at least three consecutive respiratory cycles. This average tidal breath duration was then used as the reference interval for capturing UES mean resting pressure and UES contractile integral (UES-CI) within 30 s of the beginning (UES-CI beginning) of the study during landmark measures, as well as at the end of the study (UES-CI end) within 10 s prior to catheter removal. The MedTronic smart mouse feature with rectangular box analysis was used to calculate UES mean resting pressure (mm Hg) and UES-CI (mm Hg-cm-s) by positioning the box from the proximal to the distal limit of the UES across the length of the subject’s tidal breath duration (Fig. 2).

**Fig. 2.**
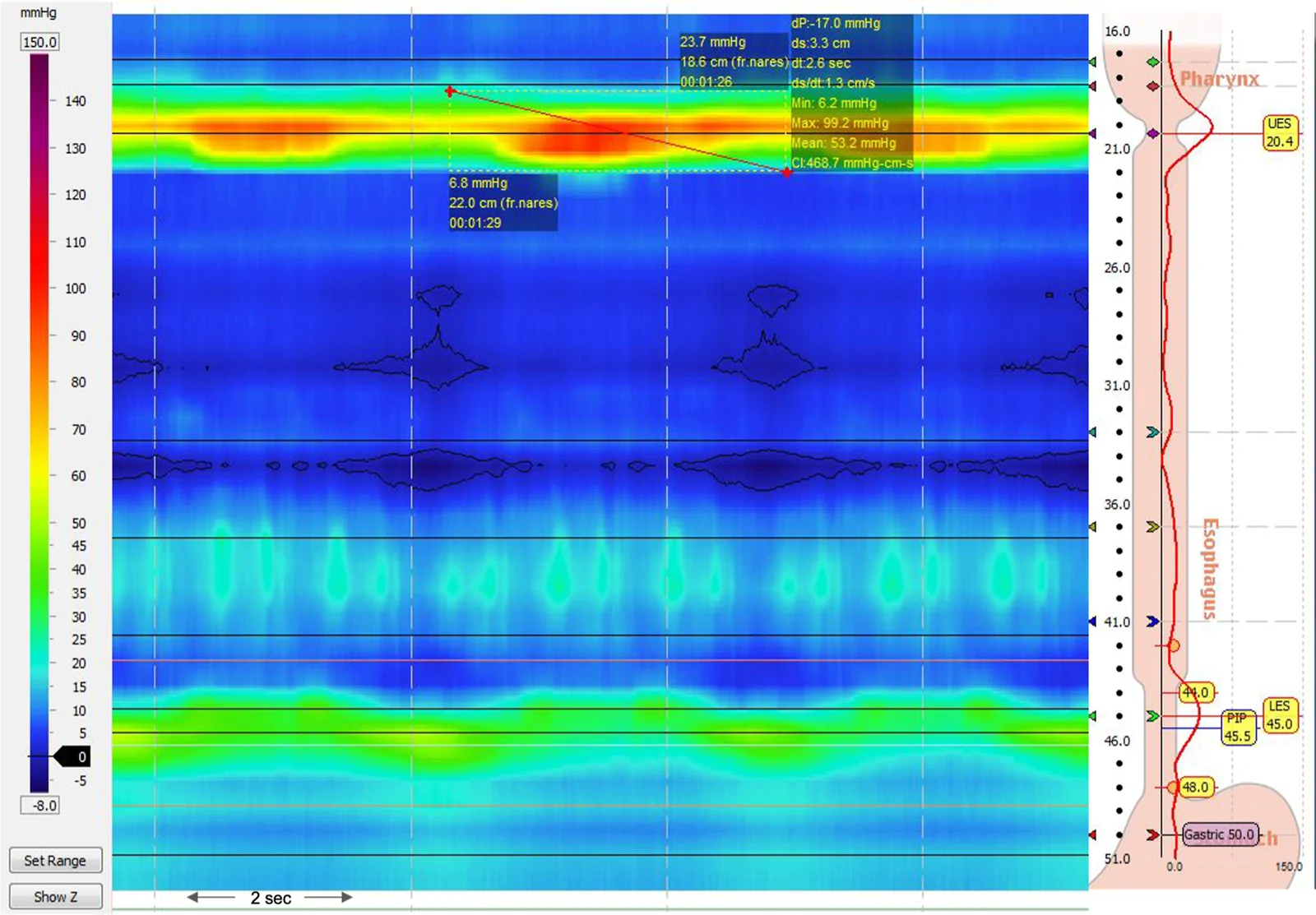
Representative image of manual measurement of UES mean pressure and UES-CI using the smart mouse feature. The start and end points of the box represent the average duration of the person’s tidal breathing respiratory cycle.

Additional esophageal pressure measures were collected from the Medtronic ManoView ESO 3.3 manometry reports including percent intact swallows, distal contractile integral (DCI) mean, esophageal mean wave amplitude, distal latency, LES mean and median basal pressures, and average UES mean residual pressure. Esophageal and LES measures are reported as an average across 10 swallows, while UES measures were collected at 30 s after catheter placement and again at the end of study. The 30 s values were only used for within-study landmark comparison to assess changes from immediately following catheter insertion to end of study conditions and were not used for diagnostic interpretation under Chicago Classification 4.0 [22]. UES mean residual pressure represents an average across 10 swallows. The UES landmark measures analyzed in this study were extracted separately by trained study personnel (S.S.) using the ManoView smart mouse protocol. HRM studies were interpreted as part of routine clinical care by attending specialists in gastroenterology, otolaryngology, or general surgery, in accordance with Chicago Classification version 4.0 criteria [22]. Study diagnoses were also collected from manometry reports based on physician assessment using Chicago Classification 4.0 criteria [22].

### Statistical Analysis

Linear regression was used to model the relationships between anxiety (STAI, HR, GSR), pain (SF-MPQ and VAS), and pressures of the esophagus, UES, and LES at rest and during swallowing. Independent covariates included (1) SF-MPQ and VAS; (2) STAI sum score (3) HR before catheter insertion (HR); and (4) average GSRslope during catheter insertion. Dependent variables included measures of UES function (i.e., UES mean residual, UES-CI), LES function (i.e., LES mean and median basal pressure), and esophageal motility (i.e., DCI mean and esophageal mean wave amplitude).

A familywise alpha of *p* < 0.05 defined statistical significance. No adjustments were used to correct for multiple comparisons since this was an exploratory study. Adjusted R^2^ were used as standardized effect sizes and interpreted as follows: small (0.05–0.16), medium (0.17–0.40), large (>0.40) [23]. As a preprocessing step, linearity, homogeneity of variance, normality of residuals, and outliers were visually inspected prior to running each regression model. Analyses were performed using R version 4.2.3 (R Core Team, 2023)

## Results

### Patient Demographics

Patients included 19 males and 17 females who had an average age of 50.8 years. Patients reported relatively low pre-procedural anxiety, as indicated by STAI (mean = 13.1, IQR of 7–18). They also reported pain (VAS pain score mean *=* 38.4) and discomfort (SF-MPQ mean = 10.6) during the procedure. Nearly half of participants had a normal manometry exam (Table 1). Among those with abnormal findings, EGJ outflow disorders were the most common diagnosis. Average UES mean basal pressure decreased from beginning to the end of exam.

**Table 1.** Patient demographics, summary of patient-reported measures, and manometric characteristics

​	Mean (SD)	Range
Age	50.8 (17.3)	23–82
Sex (% male)	53	​
Patient-reported outcomes	​	​
STAI^1^ sum (6−24)	13.1 (2.1)	7–18
VAS (0−100)^2^	38.4 (23.4)	0–83
SF-MPQ (0−45)^3^	10.6 (8.1)	0–28
Manometry diagnosis, *n* (%)	​	​
Normal	17 (47)	​
EGJ outflow obstruction^4^	14 (39)	​
Ineffective esophageal motility^5^	4 (11)	​
Other^6^	1 (3)	​
Tidal breath duration, s	3.9 (1.5)	1.5–8.22
Intact swallows (%)	79.6	0–100
Mean change (end – 30 s), mm Hg	−5.1 (20.7)	−55.6–39.5

^1^ STAI.

^2^ VAS.

^3^ SFMPQ.

^4^ Includes one achalasia-spectrum case, two studies with concurrent jackhammer features, and three qualified as possible or requiring clinical correlation.

^5^ Ineffective esophageal motility (>70% ineffective swallows or ≥50% failed peristalsis).

^6^ Hypercontractile (jackhammer) esophagus with normal integrated relaxation pressure.

### Association between Patient Measures and UES and LES HRM Outcome Measures

There was a statistically significant negative relationship between UES mean residual pressure with GSRslope and VAS. This signifies that lower UES residual pressures were associated with higher rates of physiologic response to catheter insertion and higher pain during this part of the procedure. However, there were no statistically significant relationships between patient anxiety and pain with UES-CI start of protocol, UES-CI end of protocol, or UES-CI end of protocol controlling for UES-CI start of protocol (Table 2).

**Table 2.** Estimate linear regression coefficients (β 95% CI) for the relation of upper esophageal HRM measures to STAI collected prior to start of the HRM protocol, physiologic Indicators of stress (i.e., GSRslope and HR) collected during manometer insertion, and patient-reported discomfort during manometer insertion (VAS and SF-MPQ)

Covariates	UES-CI start of protocol (30 s)	*p* value	UES-CI end of protocol	*p* value	UES-CI controlling for start	*p* value	UES mean residual	*p* value
	β (95% CI)		β (95% CI)		β (95% CI)		β (95% CI)	
Before start of protocol								​
STAI sum	1.91 (−1.36 to 5.17)	0.24	0.30 (−2.43 to 3.03)	0.82	−0.36 (−2.93 to 2.21)	0.78	−0.03 (−3.02 to 2.96)	0.99
During catheter insertion								​
HR, BPM	0.36 (−0.16 to 0.88)	0.17	0.22 (−0.21 to 0.66)	0.30	0.10 (−0.31 to 0.52)	0.61	−0.10 (−0.57 to 0.36)	0.66
GSRslope (%)^1^	−3.51 (−12.23 to 5.21)	0.42	2.37 (−4.79 to 9.54)	0.51	3.65 (−2.95 to 10.25)	0.27	−9.09 (−16.10 to −2.09)*	0.01*
Reported following protocol completion								​
VAS (0 to 100)	0.02 (−0.29 to 0.33)	0.89	−0.09 (−0.34 to 0.16)	0.48	−0.10 (−0.33 to 0.14)	0.41	−0.27 (−0.53 to −0.02)*	0.04*
SF-MPQ (0 to 45)	0.06 (−0.87 to 0.99)	0.89	−0.02 (−0.78 to 0.74)	0.96	−0.04 (−0.75 to 0.66)	0.90	−0.24 (−1.26 to 0.42)	0.32

*< 0.05; **<0.01.

^1^ Maximum slope of conductance % change.

Patient measures were also not generally associated with LES pressures (Table 3). Notably, pre-procedure HR was inversely associated with LES median basal pressure (*p* = 0.01), explaining approximately ∼15.5% of variance in this outcome (Table 3). The adjusted R^2^ values were generally low, suggesting that while these associations exist, they explain only a small portion of the variance in these outcomes.

**Table 3.** Estimate linear regression coefficients (β 95% CI) for the relation of lower esophageal HRM measures to STAI collected prior to start of the HRM protocol, physiologic indicators of stress (i.e., GSRslope and HR) collected during manometer insertion, and patient-reported discomfort during manometer insertion (VAS and SF-MPQ)

Covariates	LES mean basal pressure	*p* value	LES median basal pressure	*p* value
	β (95% CI)		β (95% CI)	
Before start of protocol				
STAI sum	1.41 (−1.96 to 4.78)	0.40	1.27 (0.02 to 2.52)	0.05*
During catheter insertion				
HR, BPM	−0.50 (−1.02 to 0.02)	0.06	−0.26 (−0.45 to −0.07)	0.01**
GSRslope (%)^1^	6.20 (−1.02 to 0.02)	0.16	0.74 (−2.75 to 4.22)	0.67
Reported following protocol completion				
VAS (0 to 100)	−0.17 (−0.48 to 0.15)	0.29	−0.06 (−0.18 to 0.06)	0.35
SF-MPQ (0 to 45)	−0.50 (−1.43 to 0.44)	0.29	−0.18 (−0.55 to 0.18)	0.31

*< 0.05; **<0.01.

^1^ Maximum slope of conductance % change.

### Association between Patient Measures and Esophageal HRM Outcome Measures

Finally, only the relationship between SF-MPQ and overall esophageal motility was statistically significant. As SF-MPQ increased, the percentage of intact swallows decreased. Notably, SF-MPQ explained ∼14.7% of the variance in percentage of intact swallows (*p* = 0.012). These esophageal results suggest that patients with higher pain during the procedure were more likely to have fewer successful swallows but that this pain does not associate with function on other esophageal motility measures (Table 4)**.**

**Table 4.** Estimate linear regression coefficients (β 95% CI) for the relation of esophageal HRM measures to STAI collected prior to start of the HRM protocol, physiologic indicators of stress (i.e., GSRslope and HR) collected during manometer insertion, and patient-reported discomfort during manometer insertion (VAS and SF-MPQ)

Covariates	Intact swallows (%)	*p* value	DCI Mean	*p* value	Esophageal mean wave amplitude	*p* value	Distal latency (*n* = 35)	*p* value
	β (95% CI)		β (95% CI)		β (95% CI)		β (95% CI)	
Before start of protocol								
STAI sum	4.71 (0.13 to 9.29)	0.04*	151.3 (−229.8 to 532.5)	0.43	2.42 (−4.58 to 9.41)	0.49	0.04 (−0.13 to 0.21)	0.64
During catheter insertion								
HR, BPM	−0.18 (−0.96 to 0.60)	0.65	−35.2 (−95.7 to 25.4)	0.25	−0.53 (−1.65 to 0.59)	0.34	0.02 (−0.01 to 0.05)	0.14
GSRslope (%)^1^	0.87 (−11.96 to 13.71)	0.89	−155.7 (−1171.9 to 860.6)	0.76	0.43 (−18.2 to 19.1)	0.96	0.20 (−0.24 to 0.64)	0.35
Reported following protocol completion								
VAS (0 to 100)	−0.36 (−0.79 to 0.08)	0.11	−11.7 (−47.4 to 24.0)	0.51	−0.10 (−0.76 to 0.56)	0.76	0.00 (−0.01 to 0.02)	0.60
SF-MPQ (0 to 45)	−1.62 (−2.85 to 0.38)	0.01*	−38.4 (−148.9 to 72.2)	0.49	−0.41 (−2.44 to 1.63)	0.69	0.03 (−0.01 to 0.08)	0.16

*< 0.05; **<0.01.

^1^ Maximum slope of conductance % change.

## Discussion

Overall, we saw few significant relationships between HRM outcomes and patient-reported anxiety and discomfort during the procedure. However, two small but statistically significant relationships emerged, one between UES mean residual pressure and VAS score and another between UES mean residual pressure and slope of GSR conductance, as a measure of physiologic response to pain. Contrary to our original hypotheses, these relationships were inversely proportional. Similar patterns were observed at the LES, where LES median basal pressure and HR were inversely related. Together, these findings suggest that higher physiologic response to pain and/or anxiety may be associated with lower sphincter pressures. Given the number of exploratory comparisons performed, significant results should be interpreted as hypothesis-generating and will need further confirmation in prospective studies. Even though these effects are small, they contribute to a growing body of research on how pain and anxiety relate to esophageal function. Importantly, our results indicate that in this sample of patients undergoing routine HRM, underlying anxiety and discomfort showed limited association with manometric outcomes.

There is conflicting evidence regarding the degree to which patient stress and anxiety during manometry affect HRM. Similar to previous studies, our findings indicate a decrease in overall UES mean pressure over the course of the procedure, suggesting that the acute stress of insertion transiently increases esophageal contraction [24]. This supports the use of baseline resting pressures taken toward the end of the protocol. Unlike previous studies linking mood symptoms to esophageal pressures, we saw no relation between DCI mean or LES pressures in relation to patient anxiety before the study [25, 26]. The relatively low anxiety levels in our sample may partly account for this, and future research might benefit from including participants’ psychiatric diagnoses. It is also possible that severity of disease and HRM findings of only certain esophageal disorders are associated with increased anxiety [27]. Additionally, prior studies linking induced stress with elevated pressures primarily involved healthy, young volunteers whereas our sample consisted of older participants with dysphagia [24].

Our findings expand on previous research by suggesting that UES residual pressure, a measure of relaxation during bolus passage from the pharynx into the esophagus, may be modestly impacted by patient anxiety and physiologic response to HRM. Elevated residual pressures may indicate incomplete relaxation of the UES, contributing to dysphagia symptoms [28]. The inverse relations seen may reflect behavioral adaptation occurring due to procedural discomfort but not necessarily reflecting changes in more stable baseline pressures. In contrast, lower esophageal basal tone is more stable across physiologic states and was only mildly associated with patient HR and anxiety possibly reflecting measurement artifact or incomplete adaptation period. Larger studies that control for additional confounding factors, such as the clinical indication for HRM, medication use, duration of symptoms, comorbidities, and neurodevelopmental differences (e.g., attention-deficit/hyperactivity disorder or autism spectrum condition), which may shape the psychosensory experience of the procedure, may help provide further insight into these interactions.

Overall, our results showed little association between pain and anxiety with resting pressures or more peristaltic metrics. However, UES mean residual pressure during swallowing was inversely associated with pain (VAS) and physiologic arousal (GSRslope). Taken together, these exploratory findings support the stability of HRM measures even in patients who experience discomfort during the procedure. While some patients may experience transiently increased UES pressure following manometer insertion, our findings indicate that these responses do not substantially correlate with patient-reported or physiologic pain and anxiety indicators.

A notable proportion of patients in our study presented with normal HRM findings, which could explain the limited associations observed. Importantly, a normal esophageal HRM does not indicate normal swallowing. Dysphagia is a symptom, as opposed to swallowing dysfunction, which relates to objective findings of abnormal deglutitive function on instrumental swallow studies [29], and sensory-based presentations, such as the functional dysphagia with maladaptive muscle tension and oral-phase behaviors described by Kang et al. [30], constitute true swallowing dysfunction that is not readily captured by motor-based assessments. Likewise, age-related esophageal changes may not meet Chicago Classification thresholds yet still contribute to symptoms, particularly when compounded by factors such as xerostomia. Because our dataset comprised esophageal HRM alone, without oropharyngeal, endoscopic, or radiographic correlates, we cannot characterize overall swallowing function in participants with normal manometry. Finally, milder impairment itself may contribute to heightened pain and anxiety during HRM. Better procedural tolerability has been reported in patients with clinician-rated moderate-to-severe impairment on videofluoroscopy than in those with milder impairment [1], possibly reflecting greater motivation to undergo testing or coexisting sensory deficits in those with greater impairment.

Our study has multiple limitations including small sample size, nature of a secondary analysis, and a limited anxiety and diagnostic distribution. Because participants were recruited consecutively from routine care pathways across three specialties, dysphagia was established by the referring clinician rather than by a standardized assessment, and instrumental swallowing outcomes (e.g., penetration-aspiration scale scores) were not available across the cohort. An important caveat is that diagnostic heterogeneity and pain- and anxiety-related effects cannot be disentangled in this dataset. Underlying motility disorders directly alter the esophageal pressure measures modeled as outcomes, and our sample size precluded diagnosis-stratified or diagnosis-adjusted analyses. Between-diagnosis variability may therefore have masked true associations with pain- and anxiety-related indicators or contributed to those we observed. Accordingly, our findings should be interpreted as hypothesis-generating. Additionally, although no association between the VR intervention and baseline anxiety or discomfort during the procedure were found, some patients with high procedural anxiety declined to participate due to concerns about additional VR-related stress [18]. A broader anxiety distribution and inclusion of patients on various psychotropic medications might reveal additional associations. Additionally, physiologic indicators were collected during HRM catheter insertion, which may not capture the most relevant stress response phase in the esophagus. Also, since UES measures were obtained in the upright position at the end of the study, positional effects on UES resting pressure may have contributed to the observed decrease, in addition to adaptation following insertion. Furthermore, UES contractile activity is also modulated reflexively by esophageal events. Esophageal-UES contractile reflexes elicited by esophageal distension or reflux are well documented as a protective response and could elevate UES-CI in patients with dysmotility or outflow obstruction independently of pain or anxiety. Because our models did not adjust for manometric diagnosis, reflexive UES responses represent an additional potential confounder of the UES-CI findings. Lastly, given the secondary nature of this analysis, further, large-scale prospective research with sufficient representation of individual diagnostic categories is warranted to confirm these findings and elucidate the mechanisms linking emotional distress with esophageal function. Future research could benefit from longer, continuous physiologic monitoring throughout the study not just during probe insertion.

## Conclusion

In this exploratory study, patient-reported anxiety and pain during HRM were generally low and showed limited association with esophageal manometry outcomes. Although small inverse relations were observed between UES residual pressure and patient-reported pain, as well as between LES median basal pressure and physiologic arousal, most sphincter and peristaltic measures were unaffected by baseline anxiety, sympathetic activation during the procedure, and subjective discomfort. These findings suggest that in this sample, patient-reported and objective measures of pain and anxiety have modest and likely clinically insignificant relations with manometric outcomes, supporting the robustness of HRM validity even in patients who experience discomfort during the procedure. However, given the limited anxiety range in this sample and high proportion of patients with normal HRM findings, larger prospective studies with broader anxiety severity, continuous physiologic monitoring, and additional clinical covariates are needed to clarify potential interactions between emotional distress and esophageal function.

## Statement of Ethics

This study was performed in accordance with the Declaration of Helsinki. This human study was approved by Weill Cornell Institutional Review Board − approval: IRB 20-01021300-16. The study’s clinical trial registration number is NCT05218967 registered with https://clinicaltrials.gov/study/NCT05218967. Participant registration took place from Feb-2022 to Aug-2022. All adult participants provided written informed consent to participate in this study.

## Conflict of Interest Statement

Anaïs Rameau owns equity of Perceptron Health, Inc., is an advisor for SoundHealth Systems, Inc., and Pentax Medical, and is a founder of VAST Health LLC. The other authors have no conflicts of interest to declare.

## Funding Sources

Anaïs Rameau was supported by a Paul B. Beeson Emerging Leaders Career Development Award in Aging (K76 AG079040) from the National Institute on Aging and by the Bridge2AI award (OT2 OD032720) from the NIH Common Fund.

## Author Contributions

A.R. and J.A.C. conceived the study. S.S. and I.P. performed the data collection and curation. S.S. completed the investigation and wrote the original draft. J.A.C. aided in the analysis. A.R. supervised the project. A.R., S. S., J.A.C., V.M., I.P., P.K., A.S., R.Z., and F.S.S. discussed the results and contributed to the final manuscript.

## Data Availability

All data generated or analyzed during this study are included in this article. Further inquiries can be directed to the corresponding author.

## References

[1] Knigge MA , Marvin S, Thibeault SL. Safety and tolerability of pharyngeal high-resolution manometry. Am J Speech Lang Pathol. 2019;28(1):43–52. 10.1044/2018_AJSLP-18-0039 30515521

[2] Knigge MA , Thibeault S, McCulloch TM. Implementation of high-resolution manometry in the clinical practice of speech language pathology. Dysphagia. 2014;29(1):2–16. 10.1007/s00455-013-9494-5 PMC4201623 24233810

[3] Omari TI , Ciucci M, Gozdzikowska K, Hernández E, Hutcheson K, Jones C, . High-Resolution pharyngeal manometry and impedance: protocols and metrics-recommendations of a high-resolution Pharyngeal Manometry International Working Group. Dysphagia. 2020;35(2):281–95. 10.1007/s00455-019-10023-y 31168756

[4] Baldwin DL , Puckett Y. Esophageal Manometry. [Updated 2022 Sept 26]. In: StatPearls [Internet]. Treasure Island (FL): StatPearls Publishing; 2022. Available from: https;//www.ncbi.nlm.nih.gov/sites/books/NBK559237/32644663

[5] Oh JE , Huang L, Takakura W, Khuu K, Wang J, Kowalewski E, . Safety and tolerability of high-resolution esophageal manometry in children and adults. Clin Transl Gastroenterol. 2023;14(5):e00571. 10.14309/ctg.0000000000000571 PMC10208713 36854056

[6] Wahba G , Neshkova E, Jutras G, Liu Chen Kiow J, Clément M, Willems P, . Lidocaine before esophageal manometry and ambulatory pH monitoring: a randomized controlled trial. Neuro Gastroenterol Motil. 2021;33(12):e14167. 10.1111/nmo.14167 33969923

[7] Walamies MA . Perception of esophageal manometry. Dis Esophagus. 2002;15(1):46–9. 10.1046/j.1442-2050.2002.00224.x 12060042

[8] Anderson KO , Dalton CB, Bradley LA, Richter JE. Stress induces alteration of esophageal pressures in healthy volunteers and non-cardiac chest pain patients. Dig Dis Sci. 1989;34(1):83–91. 10.1007/BF01536159 2910684

[9] Cook IJ , Dent J, Shannon S, Collins SM. Measurement of upper esophageal sphincter pressure. Effect of acute emotional stress. Gastroenterology. 1987;93(3):526–32. 10.1016/0016-5085(87)90915-2 3609662

[10] Lee HS , Noh CK, Lee KJ. The effect of acute stress on esophageal motility and gastroesophageal reflux in healthy humans. J Neurogastroenterol Motil. 2017;23(1):72–9. 10.5056/jnm16119 PMC5216637 28049863

[11] Mittal RK , Stewart WR, Ramahi M, Chen J, Tisdelle D. The effects of psychological stress on the esophagogastric junction pressure and swallow-induced relaxation. Gastroenterology. 1994;106(6):1477–84. 10.1016/0016-5085(94)90400-6 8194692

[12] Rubin J , Nagler R, Spiro HM, PILOT ML. Measuring the effect of emotions on esophageal motility. Psychosom Med. 1962;24:170–6. 10.1097/00006842-196203000-00007 14494912

[13] Young LD , Richter JE, Anderson KO, Bradley LA, Katz PO, McElveen L, . The effects of psychological and environmental stressors on peristaltic esophageal contractions in healthy volunteers. Psychophysiology. 1987;24(2):132–41. 10.1111/j.1469-8986.1987.tb00265.x 3602264

[14] Hjermstad MJ , Fayers PM, Haugen DF, Caraceni A, Hanks GW, Loge JH, . European Palliative Care Research Collaborative (EPCRC). Studies comparing Numerical Rating Scales, Verbal Rating Scales, and Visual Analogue Scales for assessment of pain intensity in adults: a systematic literature review. J Pain Symptom Manage. 2011;41(6):1073–93. 10.1016/j.jpainsymman.2010.08.016 21621130

[15] Cochrane Central Editorial Service, Dümmler D, Eck S, Hapfelmeier A, Fomenko A, Aktürk Z, . State-Trait Anxiety Inventory (STAI) for detecting anxiety disorders in adults. Cochrane Database Syst Rev. 1996;2025(12). 10.1002/14651858.CD015458 PMC12715840 41416518

[16] Loggia ML , Juneau M, Bushnell CM. Autonomic responses to heat pain: heart rate, skin conductance, and their relation to verbal ratings and stimulus intensity. Pain. 2011;152(3):592–8. 10.1016/j.pain.2010.11.032 21215519

[17] Papanikolaou F , Nedergaard RB, Drewes AM, Benthien KS. Electrodermal activity: the potential of psychophysiology in pain assessment. Psychophysiology. 2026;63(3):e70276. 10.1111/psyp.70276 41793211

[18] Palte I , Stewart S, Rives H, Curtis JA, Enver N, Tritter A, . Virtual reality for pain management during high-resolution manometry: a randomized clinical trial. Laryngoscope. 2024;134(3):1118–26. 10.1002/lary.30914 PMC10818016 37497865

[19] Marteau TM , Bekker H. The development of a six-item short-form of the state scale of the Spielberger State-Trait Anxiety Inventory (STAI). Br J Clin Psychol. 1992;31(3):301–6. 10.1111/j.2044-8260.1992.tb00997.x 1393159

[20] Melzack R . The short-form McGill Pain Questionnaire. Pain. 1987;30(2):191–7. 10.1016/0304-3959(87)91074-8 3670870

[21] Sanchez-Comas A , Synnes K, Molina-Estren D, Troncoso-Palacio A, Comas-González Z. Correlation analysis of different measurement places of galvanic skin response in Test groups facing pleasant and unpleasant stimuli. Sensors. 2021;21(12):4210. 10.3390/s21124210 PMC8234095 34205302

[22] Yadlapati R , Kahrilas PJ, Fox MR, Bredenoord AJ, Prakash Gyawali C, Roman S, . Esophageal motility disorders on high-resolution manometry: chicago classification version 4.0(©). Neuro Gastroenterol Motil. 2021;33(1):e14058. 10.1111/nmo.14058 PMC8034247 33373111

[23] Gaeta L , Brydges CR. An examination of effect sizes and statistical power in speech, Language, and hearing research. J Speech Lang Hear Res. 2020;63(5):1572–80. 10.1044/2020_JSLHR-19-00299 32343919

[24] Rezende DT , Herbella FA, Silva LC, Panocchia-Neto S, Patti MG. Upper esophageal sphincter resting pressure varies during esophageal manometry. Arq Bras Cir Dig. 2014;27(3):182–3. 10.1590/S0102-67202014000300005 PMC4676376 25184767

[25] Dias PN , Neto RM, Herbella FA, Zugman A, Schlottmann F, Patti MG. Psychiatric symptoms and the upper esophageal sphincter. Surgery. Gastroenterol Oncol. 2020;25:121.

[26] Raja S , Palchaudhuri S, Stein E, Nandwani M, Lynch K, Dhalla S, . Tu1164 upper esophageal sphincter pressure does not correlate with anxiety, depression or somatization. Gastroenterology. 2015;148(4):S-808.

[27] Lan QL , Lin XX, Wang Y, Xu BB, Shu KY, Zhang XJ. The relationship between upper esophageal sphincter pressure and psychological status in patients with globus sensation. Int J Gen Med. 2021;14:8805–10. 10.2147/IJGM.S337165 PMC8630375 34858048

[28] Jones CA , Hammer MJ, Hoffman MR, McCulloch TM. Quantifying contributions of the cricopharyngeus to upper esophageal sphincter pressure changes by means of intramuscular electromyography and high-resolution manometry. Ann Otol Rhinol Laryngol. 2014;123(3):174–82. 10.1177/0003489414522975 PMC4128397 24633943

[29] Rameau A , Katz P, Andreadis K, Drenis S, Joseph IT, Tran A, . Clarifying inaccurate terminology: the important difference between Dysphagia and swallowing dysfunction. Foregut. 2022;2(1):11–7.

[30] Kang CH , Zhang N, Lott DG. Muscle tension dysphagia: contributing factors and treatment efficacy. Ann Otol Rhinol Laryngol. 2021;130(7):674–81. 10.1177/0003489420966339 33090008

[31] Baldwin DP . Yana. Esophageal manometry. StatPearls. 2022. 32644663

